# Triple-Tracer Sentinel Node Mapping: Maximizing Detection, Minimizing Dissection

**DOI:** 10.3390/life15121839

**Published:** 2025-11-29

**Authors:** Daniel Alin Cristian, Bogdan Popescu, Cristian Valentin Toma, Sertaç Ata Güler, Adrian Bordea, Emil Popa, Draga-Maria Mandi, Bianca Maria Floarea, Răzvan-Valentin Scăunaşu

**Affiliations:** 1 Faculty of General Medicine, Carol Davila University, 050474 Bucharest, Romania; daniel.cristian@umfcd.ro (D.A.C.); dr.bpopescu@gmail.com (B.P.);; 2 Department of General Surgery, “Colţea” Hospital, 030167 Bucharest, Romania; 3 Department of ENT, “Colţea” Hospital, 030167 Bucharest, Romania; 4 Department of Urology, “Prof. Dr. Theodor Burghele” Hospital, 061344 Bucharest, Romania; 5 Department of General Surgery, Medical Faculty, Kocaeli University, 41380 Kocaeli, Turkey

**Keywords:** sentinel lymph node biopsy, breast cancer, indocyanine green, triple-tracer technique, lymphatic mapping

## Abstract

Background: Sentinel lymph node (SLN) biopsy often combines technetium-99m (99mTc), indocyanine green (ICG), and methylene blue (MB), but few contemporary audits quantify the performance of each tracer when used together in routine practice. Methods: We conducted a single-center retrospective audit of 111 consecutive SLN procedures for breast cancer patients undergoing SLNB using a triple-tracer approach with technetium-99m (99mTc), indocyanine green (ICG), and methylene blue (MB). We evaluated sentinel lymph node detection rates, the number of nodes retrieved, tracer concordance, and subgroup performance (including those with mastectomy and post-neoadjuvant therapy). Results: Identification was 96.4% for 99mTc (107/111), 93.7% for ICG (104/111), and 78.4% for MB (87/111). Performance was heterogeneous (Q = 26.2, *p* < 0.001); 99mTc and ICG each outperformed MB (Holm-adjusted *p* < 0.001), while 99mTc and ICG did not differ significantly. Triple-tracer workflows were associated with higher odds of detection; cross-validated AUCs reached 0.98 for 99mTc and 0.82 for ICG. Conclusions: Technetium remains a foundational tracer for SLNB, with ICG serving as a valuable adjunct that enhances nodal visualization and overall detection efficacy, and MB adds redundancy. Triple-tracer mapping achieved the best overall nodal identification and was associated with fewer sentinel nodes excised when complete tracer concordance was observed.

## 1. Introduction

Sentinel lymph node biopsy (SLNB) remains pivotal for axillary staging in early breast cancer, reducing the need for complete axillary dissection and its associated morbidity [1,2]. Sentinel lymph node (SLN) biopsy often combines technetium-99m (99mTc), indocyanine green (ICG), and methylene blue (MB), but few contemporary audits quantify the performance of each tracer when used together in routine practice.

Traditionally, SLNB has utilized a combination of technetium-99m (99mTc) and blue dye, but this dual-tracer standard is not without drawbacks. Although technetium-99 m provides reliable preoperative and intraoperative guidance, its use is often limited by high preparation costs, the need for specialized nuclear medicine facilities, and regulatory restrictions related to radioisotope handling and radioprotection. Moreover, the availability of technetium can be inconsistent in resource-limited settings, which may delay surgery or necessitate alternative approaches. Similarly, MB carries risks of allergic reactions, inconsistent detection rates, and sometimes permanent skin staining [3,4,5].

Recently, fluorescence imaging with indocyanine green (ICG) has gained widespread use as an innovative tracer in sentinel lymph node biopsy for breast cancer. Recently, indocyanine green (ICG) fluorescence imaging has emerged as an innovative mapping tool that offers real-time lymphatic visualization with high detection rates and minimal adverse effects, positioning it as a potential replacement or adjunct to conventional tracers [6,7]. Recent comparative meta-analysis studies show ICG to be consistently superior to blue dye and at least comparable to technetium, and that combining modalities improves sensitivity [8]. Furthermore, current evidence highlights the strong performance of dual ICG and MB dye combination mapping, with triple-tracer approaches providing very high identification rates [9,10,11].

Despite the growing adoption of triple-tracer protocols—combining 99mTc, ICG, and methylene blue (MB)—there remains a paucity of real-world audits quantifying the performance and interplay of these modalities in routine breast cancer surgery. Such data are especially needed in challenging clinical settings, such as after neoadjuvant chemotherapy, where tissue changes may hinder tracer migration and increase false-negative rates.

The use of SLNB in the Post-neoadjuvant chemotherapy (post-NACT) setting remains technically challenging. Tissue alterations occur that affect or slow down the migration of tracers and cause higher false-negative rates; therefore, it is recommended to use multiple tracers, to excise more lymph nodes, or, more recently, to use target-ed axillary dissection (TAD) [1,2,12].

Breast surgery centers continue to explore optimal combinations of technetium, fluorescence, and dye tracers, either alone or in combination, to ensure accurate detection while minimizing procedural morbidity and costs.

We analyzed a consecutive series of SLNB procedures, quantifying tracer detection, and modelling performance using penalized, machine-learning, and Bayesian approaches.

We aim to provide a comprehensive, data-driven assessment of each tracer’s detection performance, evaluating workflow characteristics, and model detection outcomes using contemporary statistical methods. Our goal is to clarify the roles of technetium, ICG, and MB in modern SLN mapping and inform best practices for reliable, efficient axillary staging in breast cancer.

## 2. Materials and Methods

### 2.1. Study Design and Cohort

We retrospectively audited 111 consecutive SLNB procedures performed between January 2024 and July 2025 at the tertiary oncoplastic breast unit in “Colțea” Clinical Hospital. Data were curated from operative reports, a dedicated form for recording sentinel node data that each surgeon completes after surgery and pathology records. Variables consisted of tracer injection attempts and detection (99mTc, ICG, MB), surgical technique (conservative versus mastectomy), neoadjuvant status, number of sentinel nodes, and their status.

Patients with known allergic reactions or other situations where we were unable to use all three tracers were excluded. Similarly, patients diagnosed by vacuum-assisted biopsy or those diagnosed by excisional biopsy, where lymphatic pathways may have been altered, were excluded from the start. The analyzed lot contained 114 patients, of whom 3 cases were excluded due to incomplete data.

### 2.2. Tracer Protocols

The workflow combined 99mTc, ICG, and MB per institutional protocol.

Technetium sentinel lymph node mapping was used in the “next-day” protocol and involves injecting 74 MBq of 99mTc-nanocolloid—human serum albumin nanoparticles labeled with technetium-99m—divided into two peritumoral doses of 0.15 mL each. This radiotracer provides reliable lymphatic uptake and migration while delivering a low effective dose to the patient (approximately 0.3–0.5 mSv). Operating-room personnel remain well below population-level exposure limits (<1 mSv/year). After administration, dynamic lymphoscintigraphy confirms correct tracer deposition and early lymphatic drainage, followed by delayed static imaging to delineate lymphatic pathways and accurately localize the sentinel lymph node for intraoperative guidance.

Methylene Blue Provedye 0.5%, 2 mL was injected subdermally in the peritumoral area, in four points. In each case, preoperative marking was performed on the skin supradiacent to the tumor under ultrasound guidance. For small lesions or lesions that are not detectable by ultrasound (CDIS), a DuaLok™ (Bard™, Becton Dickinson, Franklin Lakes, NJ, USA) breast localization wire, mounted under mammographic guidance, was used to assist the surgeon in locating the pathologic site and the MB injection area. We measured the time from the moment of injection, aiming for 7 minutes for methylene blue and 3 minutes for ICG, totaling 10 minutes from the start of injection to the axillary incision.

Indocyanine green is a sterile, water-soluble dye that fluoresces in the near-infrared (NIR) spectrum. After injection, it binds to plasma proteins, allowing rapid lymphatic absorption and visualization. Verdye ICG 25 mg was reconstituted 25 mg ICG in 10 mL sterile water to obtain 2.5 mg/mL. A second dilution with distilled water was performed in the syringe to obtain a concentration of 0.5 mg/mL. For each case, 2 mL of the 0.5 mg/mL solution was used, injected subdermally, periareolar at four cardinal points.

We used OPAL1^®^ (Karl Storz SE & Co. KG, Tuttlingen, Germany) NIR/ICG technology near-infrared surgical visualization system and EuroProbe 3 (Eurorad, Chennevieres/Marne, France) system for radio-guided surgery. No selective image post-processing was applied beyond white balance. The OPAL1 NIR/ICG system is tuned to excite around 805 nm (NIR range) and detect the emission at 830 nm (the ICG signal). Practical experience has shown that the best contrast was obtained when using monochrome mode, placing the camera close to the tissues being examined (20–30 cm range).

There is always a compromise between the number of lymph nodes removed and the invasiveness of the procedure, so the number of lymph nodes excised should be tuned based on the case’s metastasis risk profile. In patients with a low risk of lymphatic metastasis, we can minimize the number removed. For cases where surgery followed neoadjuvant chemotherapy, surgeons prioritized the retrieval of multiple sentinel nodes and aimed for ≥3 nodes to mitigate false-negative risk and improve SLNB performance.

### 2.3. Statistical Analysis

The collected data were analyzed using R Foundation for Statistical Computing, R 4.5 (R Core Team, Vienna, Austria). Identification rates are reported with Wilson 95% confidence intervals. Because all tracers were evaluated within the same procedures, global comparisons used Cochran’s Q test restricted to cases in which technetium, ICG, and MB were all attempted; pairwise contrasts used McNemar tests with Holm adjustment. Detection stratifications were produced for surgery type. Detection modelling combined ridge logistic regression, bias-reduced (Firth) logistic regression, generalized additive models (GAM), and Bayesian logistic regression with triple-tracer interaction terms. The bias-reduced estimator yields finite odds ratios under complete or quasi-complete separation, which occurred in some resampled folds [13,14]. Ridge logistic regression provided L2 penalty to stabilize sparse estimates [15], while logistic GAMs analyzed potential nonlinear covariate effects, as recently recommended for breast cancer prediction [16].

## 3. Results

### Cohort Characteristics and Sentinel Node Yield

Mastectomy comprised 35 procedures (31.5%) and conservative resections comprised 76. When all three tracers concordantly detected sentinel lymph nodes, the mean number of nodes harvested was significantly lower (2.14 ± 0.35) compared to non-concordant cases (2.44 ± 0.51; *p* = 0.007), suggesting more focused mapping when all three techniques converge on the same anatomic targets (Table 1).

The overall identification (Table 2) was 96.4% (95% CI 91.1–98.6%) for technetium (107/111), 93.7% (95% CI 87.6–96.9%) for ICG (104/111), and 78.4% (95% CI 69.8–85.0%) for methylene blue (87/111). Among the 111 procedures where all three tracers were attempted, Cochran’s Q indicated heterogeneous performance (*p* ≤ 0.0001). Holm-adjusted McNemar tests showed technetium outperforming methylene blue (*p* = 0.0002) and ICG outperforming methylene blue (*p* = 0.0001), while technetium and ICG did not differ significantly (*p* = 0.4795).

Table 3 presents detection rates across three clinically relevant stratifications. Technetium-99m maintained robust performance across all groups (≥95%), and ICG showed consistently high detection (91–100%), while methylene blue exhibited greater variability (69–88%), particularly in mastectomy procedures (68.6%) compared to conservative surgery (82.9%).

Positive differences in Table 4 indicate higher detection when all three tracers were used. Triple-tracer mapping showed consistent benefits across all tracers, with the largest improvement for methylene blue. When all three tracers were deployed, detection rates increased for all tracers, with methylene blue showing the most substantial improvement.

**Figure 1:**
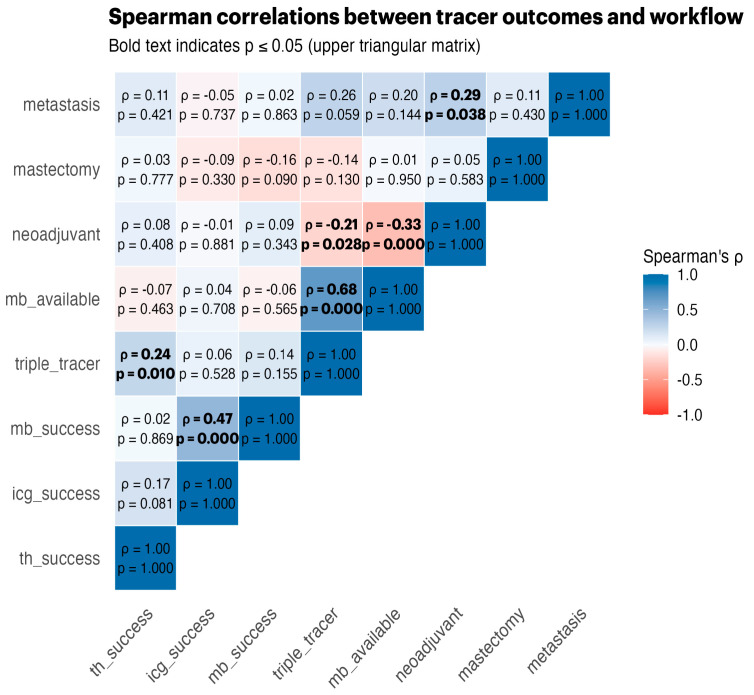
Spearman correlation heatmap for key binary tracer and workflow features.

Spearman correlations (Figure 1) revealed strong positive associations between tracer successes, triple-tracer use, and MB availability, highlighting the synergistic effects of using multiple tracers.

**Figure 2:**
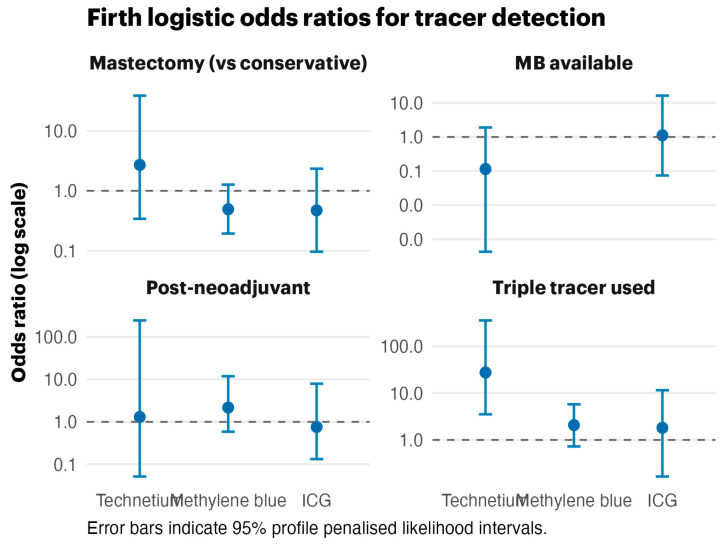
Firth logistic odds ratios across tracers with 95% profile penalized likelihood intervals. Threshold, generated by statistics program.

The Firth bias-reduced logistic regression (Figure 2) produced finite odds ratios despite near-perfect outcomes for technetium-99m. The forest plot illustrates broad confidence intervals for methylene blue and triple-tracer effects, highlighting the influence of data separation while preserving effect directionality.

Table 5 compares Firth bias-reduced and Bayesian hierarchical approaches. Both methods delivered strong discrimination for technetium-99m (AUC 0.83–0.98) and methylene blue (AUC 0.64–0.98), with Bayesian models providing a full posterior uncertainty quantification.

**Figure 3:**
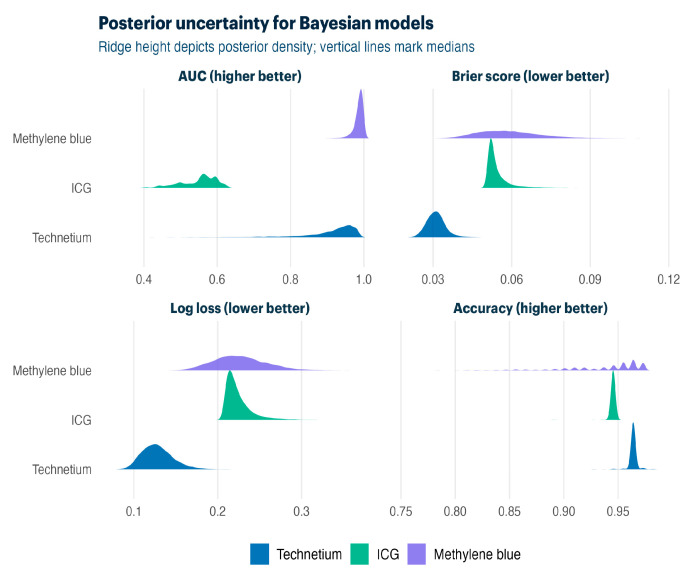
Bayesian posterior distributions for cross-validation performance metrics (AUC, Brier score, log loss, and accuracy).

Bayesian posterior distributions (Figure 3) quantify the uncertainty around model performance. Ridge plots display the median performance with spread, confirming a strong discrimination against all tracers while showing posterior uncertainty due to limited sample sizes in separate outcome scenarios.

We extracted BMI data from preoperative records for all 111 patients. The mean BMI was ± SD 27.0 ± 3.9 kg/m^2^, ranging from 18.2 to 38.6. A total of 22.5% of patients had a BMI > 30, with no missing values. Patients were categorized according to the WHO classifications: normal weight (18.5–24.9 kg/m^2^), overweight (25.0–29.9 kg/m^2^), and obese (≥30.0 kg/m^2^).

We had seven ICG failures and a mean BMI of 27.3 ± 3.7 kg/m^2^ vs. 27.0 ± 4.1 kg/m^2^ in successes (*p* = 0.83). Fluorescence maintains a >92% detection rate across normal-weight, overweight, and obese patients (Table 6). Fluorescence maintains a >92% detection rate across normal-weight, overweight, and obese patients.

## 4. Discussion

Real-world experience shows that the use of technetium best guides the surgeon in planning the operation: through a preoperative lymphoscintigraphy, which provides information on the number of lymph nodes that capture the radioactive isotope and feedback from the radiologist regarding the speed of the tracer diffusion and because it allows reliable signal detection prior to the start of the surgery, which facilitates the planning and selection of an optimal incision site. Furthermore, after the excision of the sentinel lymph node and its ex vivo measurement and confirmation, the axilla is re-evaluated in terms of radioactivity to detect any additional radioactive lymph nodes. In our opinion, this allows for a higher degree of confidence and a more limited dissection compared to dyes, which may require wider and closer exposure to ensure that no tracer-capturing lymph node is overlooked.

The use of ICG is useful and provides real-time mapping of the lymphatics, but in patients with a high body mass index, the lymphatic pathway can descend deeply in the tissues and become invisible at the axillary level. This has been our experience using LED-based detection equipment. It is possible that detection systems using laser-based light sources, which use a narrower and more focused wavelength, can highlight fluorescence at greater depths. This does not appear to affect the lymph node detection rate but rather the visualization of the lymphatic pathways and the surgeon’s orientation toward the optimal site for incision. Once the incision is made, it is possible to approach the lymph nodes more closely, allowing them to be visualized again.

Another observation derived from our experience is that the time elapsed from the injection to dye diffusion and lymph node detection is relatively short. Thus, prolonged axillary dissection times may lead to the flooding of the drainage basin and the detection of multiple lymph nodes, compared to other tracers. Contrary to these concerns that ICG use may result in the removal of excess SLNs, our data showed no evidence of an increased node excision associated with ICG detection. A mean of 2.23 ± 0.42 sentinel lymph nodes were excised per patient (median 2, range 2–3). This count remained consistent regardless of the intraoperative ICG detection status: 2.21 ± 0.41 nodes when ICG successfully identified SLNs (n = 108) versus 2.50 ± 0.55 nodes when the ICG detection failed (n = 6, *p* = 0.10). Moreover, a successful detection by all three tracers was associated with fewer nodes removed (2.15 ± 0.36, n = 71) compared to cases where fewer than three tracers were identified (2.35 ± 0.48, n = 43; *p* = 0.017), suggesting that multi-tracer concordance facilitates more targeted excision rather than indiscriminate removal.

In contrast, because MB dye tracer diffuses more slowly, in our workflow, methylene blue was injected first, giving it a longer diffusion time compared to ICG. We chose to inject MB peritumoral, which often allows it to be excised together with the resection specimen, thus enabling us to avoid known complications such as skin necrosis or tattooing. Another advantage would be the avoidance of staining of the periareolar tissue planes, which provides comfort in cases where oncoplastic surgery techniques are used and permits a better assessment of tissue vascularization.

Both overall and stratified detection rates, demonstrate concordance with recent dual and triple tracer data available in literature. A randomized study reported 100% SLN identification and the highest node yield for the triple-tracer approach (99mTc + ICG + MB) [11,17]. Meta-analyses concur that ICG is comparable to radioisotope and markedly superior to blue dye [8]. Our paired tests and model-based estimates are therefore consistent with the broader literature: raw detection odds improved approximately 12.3-fold for technetium and 1.5-fold for ICG when all tracers were available, while ridge-shrunken odds ratios (3.93 and 1.47) confirmed the same trend of effect.

Guidelines and recent evidence suggest that the combined use of technetium, fluorescence, and blue dye provides the most reliable mapping when neoadjuvant therapy or local tissue rearrangements make mapping difficult. Post-neoadjuvant performance was maintained when dual or triple tracers were attempted, echoing practice recommendations to use multiple mapping modalities after chemotherapy and to retrieve several sentinel nodes [1,2,12,18,19].

Previous contributions from our team have emphasized the importance of conservative surgery for achieving two key objectives of the intervention: preserving the mammary glandular tissue and maintaining the axillary lymphatic channels [20,21,22]. This study continues these pursuits by examining the performance characteristics of triple-tracer sentinel lymph node mapping to optimize staging accuracy while supporting high-quality surgical outcomes. Mastectomy procedures also benefited from redundancy: technetium sustained 97.3% detection, ICG maintained 92.1%, and MB, while lower at 71.1%, contributed additional mappings when fluorescence was limited. This pattern complements reports that fluorescence can recover difficult mappings and that multi-tracer strategies reduce discordance between modalities [23,24]. Dual-tracer ICG + MB series have also demonstrated excellent detection without radioisotope detection [10].

Although ICG was administered periareolar and MB peritumoral, this approach reflects their safest clinical application. Both injection routes reliably drain to the same sentinel physiological basin, and numerous anatomical and clinical studies demonstrate that superficial and deep lymphatic systems converge before reaching the axilla. The higher detection rate observed with ICG, therefore, represents a practical comparison of real-world performance rather than an isolated effect of injection site choice [25,26,27]. Accordingly, assessing tracer performance within the context of their standard clinical use provides a representative and practical comparison of detection efficacy, rather than an evaluation based on uniform injection routes that are not routinely employed in clinical practice. All of the information obtained supports the conclusion that triple-tracer concordance correlates with fewer nodes harvested (“more” targeted), leading to better clinical outcomes, fewer complications, and more efficient use of medical resources.

Limitations include the retrospective scope of a single center, the recent implementation of multiple sentinel lymph node mapping techniques, and the absence of external validation, but with the highlight that patients where lymphatic pathways may have been altered were excluded from the start.

Future work should integrate clipped-node localization and focus on performance in the NAC setting, while also exploring the economic and perioperative impacts. This consideration should take into account the real-world advantages reported for ICG-guided approaches [28].

## 5. Conclusions

This study showed that mapping sentinel lymph nodes with three markers—technetium-99m, indocyanine green fluorescence, and methylene blue—maximizes detection rates and improves the accuracy of axillary staging in breast cancer surgery. Our experience shows that technetium-99m remains the cornerstone of SLNB, offering advantages related to the preoperative planning and intraoperative guidance of axillary dissection. Indocyanine green fluorescence is robust, provides real-time lymphatic visualization with a superior safety profile and detection performance close to that of technetium, and is particularly valuable in situations where the radioisotope availability is limited or contraindicated. Methylene blue, although rarely used as a single tracer, adds redundancy and proves beneficial in technically difficult cases or when other modalities are inconclusive.

It is important to note that our results show that the combined use of all three tracers leads to greater concordance, allowing for more precise nodal excision and reducing the potential for unnecessary disease-free lymph node removal. This targeted approach can minimize surgical morbidity while maintaining a high level of oncological safety.

The benefits of three-tracer mapping were consistent across all surgical subgroups, including those undergoing mastectomy and patients who underwent surgery after neoadjuvant chemotherapy, supporting its broad applicability in diverse clinical contexts.

Despite its value as a real-world experience, limitations include its single-center, retrospective design and lack of external validation.

In summary, optimizing the selection and combination of sentinel lymph node tracers—guided by patient and procedural factors—can ensure both reliable axillary staging and improved surgical outcomes. Our results support the adoption of a tailored, multi-tracer approach as best practice for contemporary SLNBs in breast cancer.

The optimal combination of tracers for maximizing detection while minimizing morbidity and unnecessary disease-free node excision remains an area of active investigation.

## Figures and Tables

**Table 1:** Sentinel lymph node yield according to triple-tracer concordance status.

Concordance Status	Patients	Mean Nodes	SD	Median	Range
Concordant (all 3 tracers)	84	2.14	0.35	2	2–3
Non-concordant	27	2.44	0.51	2	2–3

Welch’s *t*-test: mean difference = 0.3 nodes (95% CI: 0.09–0.51), *p* = 0.007. Concordant detection (all three tracers identified nodes) is associated with fewer sentinel nodes harvested.

**Table 2:** Overall tracer detection among attempted procedures.

Tracer	Attempted	Detected	Detection Rate
Technetium-99m	111	107	96.4%
Indocyanine Green	111	104	93.7%
Methylene Blue	111	87	78.4%

**Table 3:** Detection rates stratified by surgical approach and metastasis status.

Tracer	Surgery Type	Surgery Type	Metastasis	Metastasis
Tracer	Conservative	Mastectomy	Mets−	Mets+
Technetium-99m	96.1%	97.1%	95.0%	100.0%
Indocyanine Green	94.7%	91.4%	92.5%	92.3%
Methylene Blue	82.9%	68.6%	82.5%	84.6%

All values are detection rates (%). Stratifications show consistent performance for Tc-99m and ICG, with lower MB detection in mastectomy procedures.

**Table 4:** Absolute detection rate differences with 95% cis—triple tracer delta.

Tracer	Difference	95% CI
Technetium-99m	10.8%	1.5–28.8%
ICG	2.2%	−7.0–19.5%
Methylene blue	13.4%	−4.2–34.1%

**Table 5:** Cross-validated performance metrics for Firth and Bayesian models.

Tracer	Model	AUC	Brier	Log Loss	Accuracy
Indocyanine Green	Firth (bias-reduced)	0.615	0.063	0.253	0.935
	Bayesian hierarchical	0.539	0.056	0.232	0.944
Methylene Blue	Bayesian hierarchical	0.982	0.070	0.262	0.935
	Firth (bias-reduced)	0.635	0.181	0.541	0.744
Technetium-99m	Bayesian hierarchical	0.983	0.036	0.152	0.964
	Firth (bias-reduced)	0.830	0.041	0.164	0.954

AUC = area under ROC curve; Brier = Brier score (lower is better); and Log Loss = logarithmic loss (lower is better). Bayesian hierarchical models incorporated patient-level random effects and workflow covariates.

**Table 6:** When applying the logistic regression we had an OR of 0.97 per BMI unit, (95% CI 0.86–1.09), *p* = 0.64.

BMI Category	N	ICG Detected	95% CI
Normal (<25)	42	40/42 (95.2%)	84.2–99.4%
Overweight (25–29.9)	44	41/44 (93.2%)	81.8–98.6%
Obese (≥30)	25	23/25 (92.0%)	74.0–99.0%

## Data Availability

The data presented in this study are available on request from the corresponding author. The data are not publicly available due to patient privacy and ethical restrictions.
